# Role of tau in the spatial organization of axonal microtubules: keeping parallel microtubules evenly distributed despite macromolecular crowding

**DOI:** 10.1007/s00018-016-2216-z

**Published:** 2016-04-13

**Authors:** Alix Méphon-Gaspard, Mirela Boca, Catherine Pioche-Durieu, Bénédicte Desforges, Andrea Burgo, Loic Hamon, Olivier Piétrement, David Pastré

**Affiliations:** 1 Institut National de la Santé et de la Recherche Médicale (INSERM), UMR1204, Université Evry-Val d’Essonne, Evry, 91025 France; 2 UMR 8126, CNRS, Gustave Roussy Université Paris Sud, Université Paris-Saclay, Villejuif, 94805 France

**Keywords:** Alzheimer, Tauopathy, Cytoskeleton, Neuron

## Abstract

Opposing views have been proposed regarding the role of tau, the principal microtubule-associated protein in axons. On the one hand, tau forms cross-bridges at the interface between microtubules and induces microtubule bundling in neurons. On the other hand, tau is also considered a polymer brush which efficiently separates microtubules. In mature axons, microtubules are indeed arranged in parallel arrays and are well separated from each other. To reconcile these views, we developed a mechanistic model based on in vitro and cellular approaches combined to analytical and numerical analyses. The results indicate that tau forms long-range cross-bridges between microtubules under macromolecular crowding conditions. Tau cross-bridges prevent the redistribution of tau away from the interface between microtubules, which would have occurred in the polymer brush model. Consequently, the short-range attractive force between microtubules induced by macromolecular crowding is avoided and thus microtubules remain well separated from each other. Interestingly, in this unified model, tau diffusion on microtubules enables to keep microtubules evenly distributed in axonal sections at low tau levels.

**Electronic supplementary material:**

The online version of this article (doi:10.1007/s00018-016-2216-z) contains supplementary material, which is available to authorized users.

## Introduction

Tau has been the subject of extensive studies by the past owing to its central role in many neurodegenerative diseases including Alzheimer’s disease [1–6]. However, despite the in-depth study of tau functions in neurons, both its regulatory role in axonal architecture and its contribution to neurodegeneration remain open questions. In the most popular model, tau stabilizes microtubules in axons [6–15]. During neuronal degeneration, tau is phosphorylated and possibly released from microtubules, which may then destabilize axonal microtubules [12–15]. Here, we examine whether tau is specifically designed to prevent the formation of axonal microtubule bundles. Indeed, axonal microtubules are well separated in transverse sections of mature neurons [16–20]. This alternative role for tau has already been proposed by others based on the polymer brush model [21–23] but has not truly emerged in the scientific community. Tau is rather known to form microtubule bundles via complementary dimerization in between microtubules [24–26], which is hard to reconcile with its role as microtubule spacer. In addition, a continuous repulsive layer of tau on microtubules is required to keep microtubules separated in the polymer brush model [21–23, 27], which is most probably not relevant to the conditions found in axons. This point is even more critical since tau diffuses on microtubules [28] and thus has the possibility to move away from the interface between microtubules.

Besides these diverging views, studying whether tau keeps microtubules well separated in axons makes sense for two reasons: (1) their cylindrical geometry and rigid structure make microtubules prone to collapse due to short-range (<5 nm) excluded volume interactions in the crowded cellular environment [29, 30]; (2) the organization of axonal microtubules into parallel arrays significantly increases the probability of microtubule bundling in axons. From a functional point of view, the surface of isolated microtubules is more accessible than in bundles. Keeping microtubules separated should then favor the accessibility of molecular motors to microtubule surface and thus the long-range transport of cargoes [20].

Here we propose and examine an alternative mechanistic model which reconciles tau-mediated microtubule bundling observed in vitro and the proposed physiological function of tau as a microtubule spacer. Indeed we describe how the formation of transient tau cross-bridges at the interface between parallel microtubules could lead to a very efficient separation of microtubules in axons. In addition, in this model, tau diffusion on microtubules enables an efficient separation of microtubules even at low tau levels, more in line with those found in axons.

## Results

### Analysis of the state of art and clarifications

Four points need to be clarified from the rich literature on tau and provide a basis for the present study:

1. In the literature, the fact that tau leads to the formation of microtubule bundles has been reported many times. Tau is then considered to be a positive regulator of microtubule bundling. This view stems mostly from the formation of microtubule bundles in neuronal and non-neuronal mammalian cells over-expressing tau [25, 26, 31–33]. As the spacing between microtubules in these bundles matches the length of the N-terminal domain of tau [31], this was considered as an evidence that tau can induce microtubule bundling. In line with this, the N-terminal domain of tau contains alternating clusters of positive and negative residues and can act as an antiparallel electrostatic zipper [24]. However, in electron micrographs of mature axon sections, microtubules appear as homogeneously distributed (for examples see [16–20]) and have a larger separation distance than in tau-mediated bundles (about 20 nm [32]). Along with this, the density of microtubules estimated from axonal sections in electron micrographs most frequently ranges from about 15 to 160 microtubules per µm^2^ [16, 17, 34–37]. The mean spacing between microtubules thus ranges from 79 to 260 nm. These facts indicate that tau-mediated bundling is not prevalent in mature axons. The presence of axonal microtubule bundles can nevertheless be observed at early stage during axon differentiation [34] and in the axon hillock [38] with possible neuronal functions but this is not the norm.
2. In contrast, tau may act as a polymer brush and keep microtubules separated from each other. Based on thermal movements, the unstructured N-terminal domain of tau leads to a repulsion of entropic origin between microtubules [21, 23]. The so called “polymer brush” model works as long as tau forms a continuous repulsive layer on the microtubule surface. In addition, as recently measured [28, 39], tau is not immobile on microtubules but rather diffuses along the microtubule surface (*D* = 0.15 µm^2^/s). The consequences of tau diffusion on the putative entropic repulsion occurring in the polymer brush model need to be clarified. Indeed diffusing tau can be redistributed away from the interface between microtubules. In this case, tau could no longer exert a repulsion force to keep microtubules separated. In the absence of any additional factors, only an elevated tau:tubulin molar ratio and its associated steric hindrance can prevent tau from moving away from the interface between microtubules [40], which leaves the relevancy of this model in axons questionable.
3. The tau:tubulin ratio in axons is actually poorly characterized. This is highly surprising since this information is critical to decipher the functions of tau in axons. In the literature and despite numerous publications on tau:microtubule interactions, only limited sources of data have been used to provide an estimation of the tau:tubulin molar ratio. In microtubules assembled from brain extracts [41], which may not be representative of axons, the reported tau:tubulin molar ratio was about 1:12 and 1:38 in gray and white matters, respectively. In another report [42], the tau:tubulin measured from undifferentiated to differentiated PC12 cell extracts ranges from about 1:34 to 1:5 and from 1:68 to 1:17. Tau concentration was also estimated using radioimmuno-slot-blot assay [43]. About 0.9 ng of tau per µg of proteins was found in postmortem human brain homogenates of grey matter. If we assume that there is about 30 ng of tubulin per µg of proteins in brain extract [44], the tau:tubulin molar ratio in neurons could be lower than 1:30 as grey matter contains more tubulin than white matter. In summary, all these data, although useful, do not provide a precise estimation of the specific tau:tubulin ratio in axons but rather a global estimation for neurons or brains. We therefore need to consider this point in the present study.
4. Microtubules are highly prone to form bundles under macromolecular crowding conditions. In vitro, 0.3–1 % (w/v) of PEG 35K (polyethylene glycol of 35 kDa), a neutral crowding agent, is sufficient to trigger microtubule bundling while more than about 10 % of PEG 35K are required for compacting DNA under same ionic conditions [29]. Microtubules are rigid and large cylinders with diameters about 25 nm. They thus offer a large surface for excluded-volume interactions. In vitro, microtubule bundles obtained via excluded-volume interactions under macromolecular crowding conditions are tightly packed with wall-to-wall contacts, in contrast with the regularly spaced microtubules observed in tau-mediated bundles [30]. Microtubule-based transport should most probably be impaired in such compacted structures [45], which would be detrimental for most axonal functions.

### The role of Tau 2N on microtubule bundling is biphasic

**Fig. 1:**
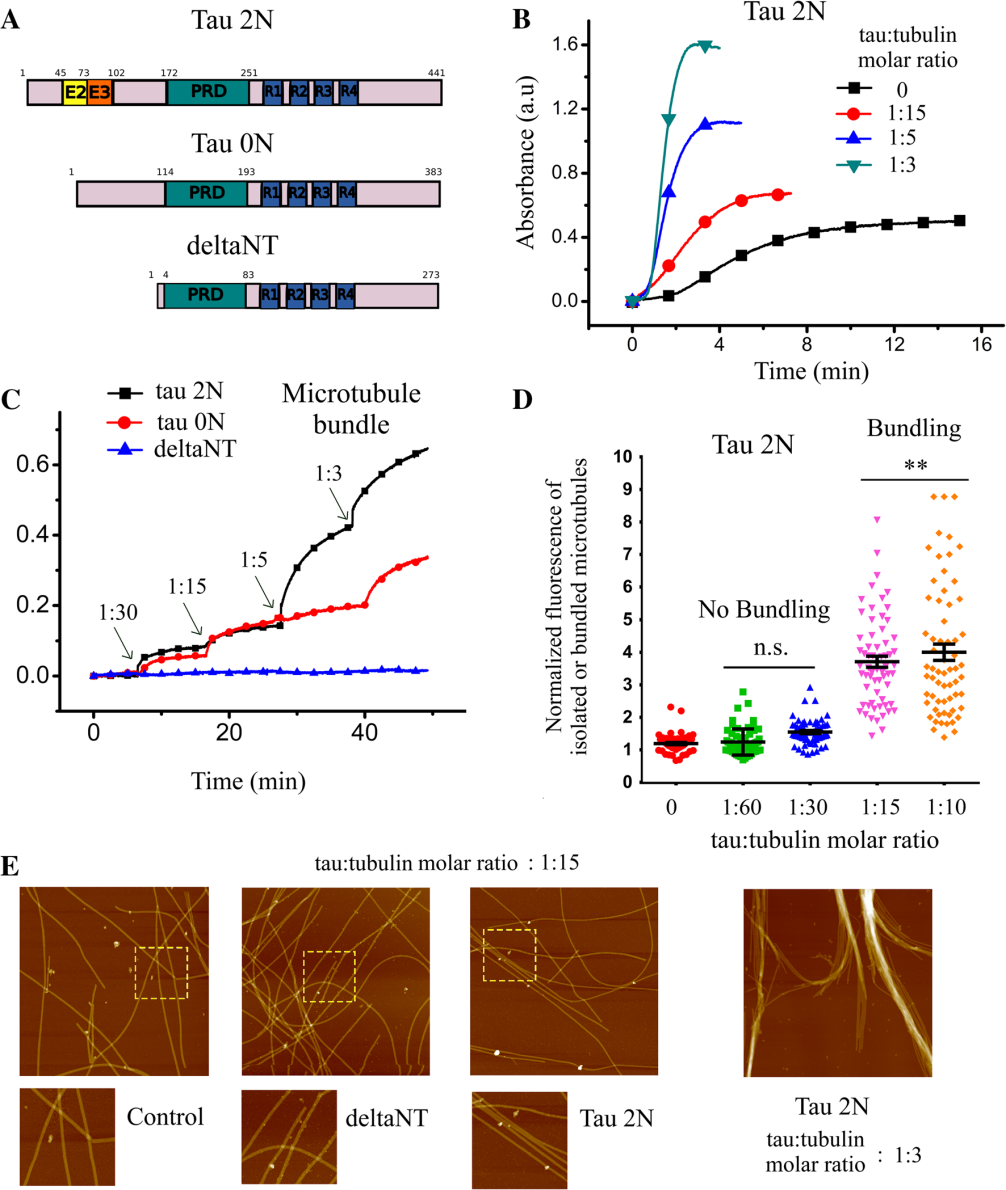
Tau leads to microtubule bundling in vitro via its N-terminal domain. **a** Schematic representation of the tau constructs used in this study. **b** Turbidimetry curves recorded in the presence of tubulin and tau 2N at indicated tau:tubulin molar ratios after raising the temperature from 5 to 37 °C. The increase of plateau value of the assembly curves in the presence of tau 2N could be the result of three putative and independent contributions: (1) increase of final microtubule mass, (2) tau aggregation, (3) tau-induced microtubule bundling [46]. We controlled that tau alone does not increase the absorbance so that the contribution of tau aggregation can be excluded. In addition, owing to the magnitude of the increase in the plateau value observed for tau:tubulin molar ratios of 1:5 and 1:3, microtubule stabilization cannot solely account for this phenomenon. Polymerization buffer: 10 mM HEPES–KOH pH 6.8, 30 mM KCl, 20 % glycerol, 1 mM EGTA, 4 mM MgCl_2_, 1 mM GTP. 20 µM tubulin were used for microtubule polymerization. **c** Microtubule bundling observed via turbidimetry measurements of taxol-stabilized microtubules after the addition of tau 2N, tau 0N and deltaNT. In the presence of taxol, only microtubule bundling contributes to the increase of absorbance. Tau 2N gradually induces microtubule bundling. DeltaNT does not induce microtubule bundling. Same buffer as **b** with 10 µM tubulin and 5 µM taxol. **d** Statistical analysis of microtubule bundling obtained from optical microscope images of taxol-stabilized microtubules deposited on mica in the presence or absence of tau 2N or deltaNT (see figures S2 for details). The increase of the normalized fluorescence intensity reveals that tau 2N induces a massive microtubule bundling at tau:tubulin molar ratio higher than 1:15. Results are mean ± SD (*n* = 60). Two-tailed *t* test, **p < 0.01. **e** High resolution imaging by atomic force microscopy shows the formation of loose microtubule bundles in the presence of tau 2N at a tau:tubulin molar ratio of 1:15. Such pattern was not observed with deltaNT. At high tau:tubulin molar ratio (1:3), tau2N leads to the formation of large bundles. Same conditions as **c** with taxol-stabilized microtubules. Scanned area: 5 × 5 µm^2^. *Lower* panels represent higher magnification images of the area corresponding to the *dashed squares*

To reassess tau-mediated bundling in vitro and the impact of the tau:tubulin molar ratio in microtubule bundling, microtubule bundling was monitored by turbidimetry measurements at 37 °C [46] in the presence or absence of tau 2N, the longest tau isoform with four microtubule-binding domains (Figs. 1a and S1). The results indicate that tau 2N triggers microtubule bundling in both taxol-stabilized and non-stabilized microtubules at moderate ionic strength (Fig. 1b, c). The onset of microtubule bundling corresponds to a tau:tubulin molar ratio of about 1:30 and microtubule bundling increases steadily at higher ratios. These results were further confirmed by optical microscopy and atomic force microscopy analyzes. They revealed the appearance of aligned microtubules and loose microtubule bundles in the presence of tau 2N (Figs. 1d, e and S2). According to the literature, tau cross-bridging at the interface between two parallel microtubules is due to the presence of alternating positive and negative charges located in the N-terminal domain of tau [24]. An electrostatic zipper mechanism is therefore sensitive to the ionic strength or to the presence of perturbing zwitterions at high concentrations. In line with this, the presence of PIPES or MES at elevated concentrations (50 mM) or monovalent salts (>75 mM) impairs microtubule bundling mediated by tau 2N (figure S3), which may explain why microtubule bundling has not been detected in some reports [47, 48], in contrast to others [24, 49, 50]. We also cannot exclude that increasing the ionic strength reduces the affinity of tau for microtubules and, in turn, also reduces the probability to form tau cross-bridges. Indeed both the formation of tau cross-bridges and the binding of tau to microtubules are partly based on electrostatic interactions.

**Fig. 2:**
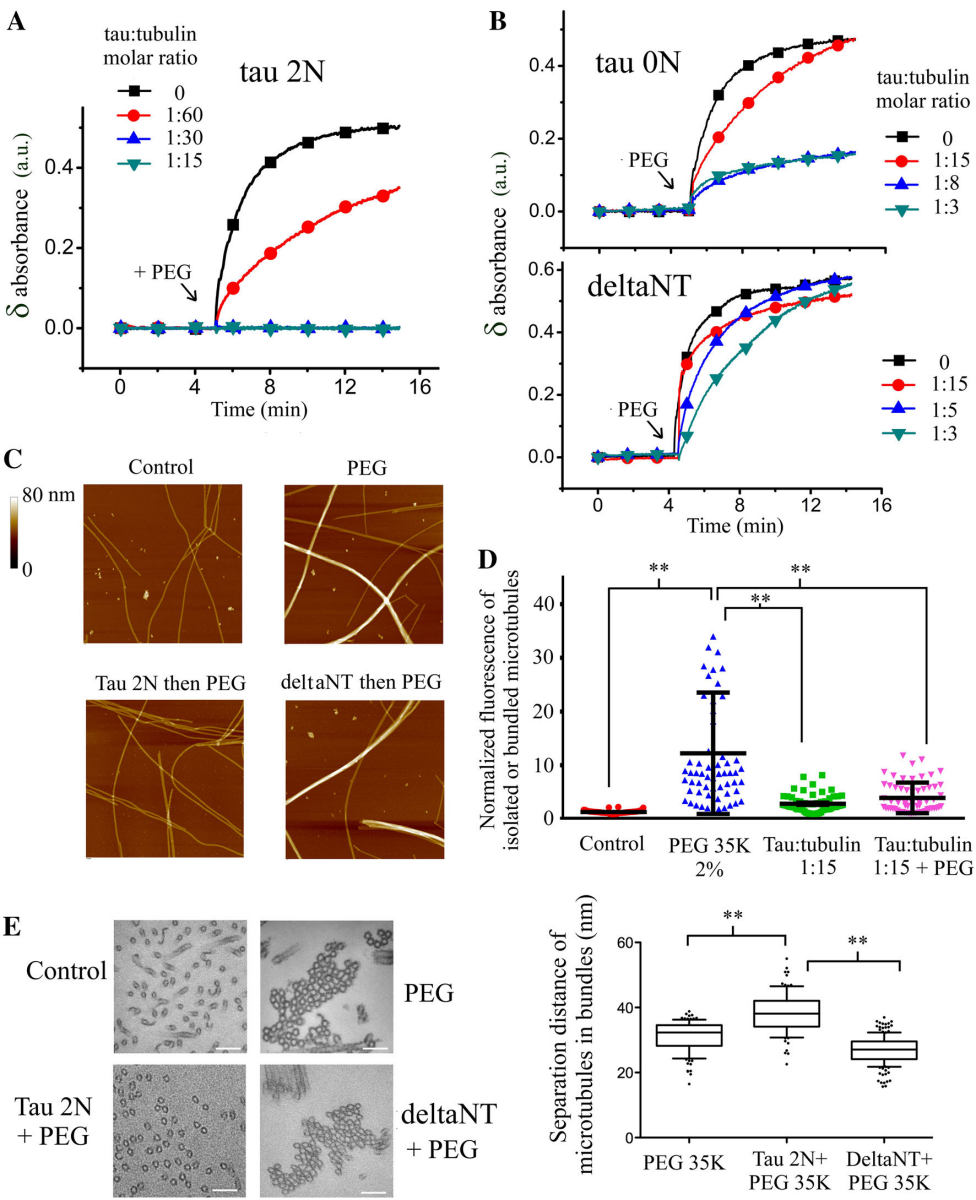
Tau antagonizes microtubule bundling under macromolecular crowding conditions. **a** Variations of absorbance recorded after the addition of PEG 35K 1 % to taxol-stabilized microtubules in the absence or presence of tau 2N at indicated concentrations. The presence of tau 2N prior to adding PEG 35K blocks microtubule bundling and so even at low tau:tubulin molar ratio (1:30). Buffer: 10 mM HEPES–KOH pH 6.8, 30 mM KCl, 20 % glycerol, 1 mM EGTA, 4 mM MgCl_2_, 1 mM GTP, 10 µM tubulin and 5 µM taxol. **b** Same experiments as **a** in the presence of tau 0N or deltaNT. DeltaNT fails to prevent microtubule bundling by PEG 35K. Tau 0N prevents microtubule bundling but to a lesser extent than tau 2N. **c** Atomic force microscopy images of taxol-stabilized microtubules after the addition of 1 % PEG 35K for 15 min. In control, PEG 35K leads to the appearance of tight microtubule bundles. In contrast with deltaNT, tau 2N inhibits the formation of tight microtubule bundles in the presence of PEG 35K 1 % for 15 min. Scanned area: 5 × 5 µm^2^. **d** Statistical analyzes of microtubule bundling from optical microcopy images of taxol-stabilized microtubules deposited on mica in the presence of tau 2N. The addition of PEG 35K 1 % leads to the formation of microtubule bundles in control. The presence of tau 2N inhibits the massive microtubule bundling induced by the addition of PEG 35K. Results are mean ± SD (*n* = 60). Two-tailed *t* test, **p < 0.01. **e** Electron micrographs reveal that microtubules exposed to 1 % PEG 35K for 15 min form tightly packed bundles. In the presence of tau 2N, microtubules failed to form tightly packed bundles after the addition of PEG 35K. In contrast, the presence of deltaNT does not prevent the formation of tightly packed bundles under macromolecular crowding conditions. *Scale bars* 100 nm. Tau:tubulin molar ratio: 1:8 for both tau 2N and deltaNT. Statistical measurements of inter-microtubule distances (*center* to *center*) inside bundles under indicated conditions in the presence of 1 % PEG 35K. The separation distance is larger in the presence of tau 2N. Interestingly, the packing of microtubule with deltaNT is tighter than in control conditions. We attributed this fact to the neutralization of the *negatively* charged C-terminal tail of tubulin by the positive residues of deltaNT, which reduces the electrostatic repulsion of microtubules. Results are mean ± SD, (*n* > 95). Two-tailed *t* test, **p < 0.01

In order to examine the putative role of tau in microtubule bundling under macromolecular crowding conditions, we used PEG 35K whose size is comparable to proteins in the cell cytoplasm (about 4–5 nm). 1 % PEG 35K is sufficient to form microtubule bundles (Fig. 2a, c). This pattern was not observed with PEG 1K because its size is not sufficient to induce significant excluded volume interactions [29]. We then analyzed whether tau 2N positively or negatively regulates the packing of microtubules under macromolecular crowding conditions. The formation of microtubule bundles was monitored by turbidimetry measurements in the presence of tau 2N after the addition of PEG 35K. The results reveal that tau 2N antagonizes the collapse of microtubules into tightly packed bundles. Importantly, at low tau-tubulin molar ratios, 1:30 and to a lesser extent at 1:60, tau 2N still antagonizes microtubule bundling (Fig. 2a). Analyzes by optical, atomic force and electron microscopies further confirm that tau 2N prevents the formation of tightly packed microtubule bundles under macromolecular crowding conditions (Fig. 2c–e).

The role of tau 2N in microtubule bundling could thus be misleading. On the one hand, in the absence of macromolecular crowding, tau 2N induces the formation of bundles at elevated tau:tubulin molar ratios. On the other hand, under macromolecular crowding conditions, tau 2N antagonizes microtubule bundling and so even at low tau 2N:tubulin molar ratios. Interestingly, at elevated ionic strength, tau 2N can no longer prevent microtubule bundling in the presence of PEG 35K (Figure S4A). As high ionic strengths also prevent microtubule bundling mediated by tau 2N in the absence of PEG 35K (Figure S3), there could be an unexpected correlation between the formation of tau cross-bridges between microtubules and the ability of tau to prevent microtubules from collapsing into tight bundles under macromolecular conditions.

### The N-terminal domain is critical for preventing microtubule bundling under macromolecular conditions at low tau:tubulin molar ratio in vitro

To explore the role of the N-terminal domain of tau 2N on microtubule bundling, we considered three tau constructs (see Fig. 1a) of different N-terminal length: tau 2N, the longest tau 4R isoform used as a control, tau 0N, the smallest isoform of tau 4R, and deltaNT, tau 4R deleted from its N-terminal domain except part of the proline rich domain which is critical for the binding of tau to microtubules [51].

In the absence of macromolecular crowding, deltaNT shows no detectable microtubule bundling activity while tau 0N could form microtubule bundles but to a lesser extent than tau 2N (Fig. 1b). DeltaNT also fails to induce the formation of loose microtubule bundles as observed with tau 2N (Fig. 1e). The propensity of the tau constructs to form microtubule bundles thus correlates positively with the length of the N-terminal domain, which emphasizes the critical role played by the N-terminal domain of tau in microtubule bundling in vitro.

Importantly, under macromolecular crowding conditions, deltaNT cannot prevent microtubule bundling (Fig. 2b, c, e) and tau 0N limits the formation of microtubule bundles under macromolecular crowding conditions to a lesser extent than 2N (Fig. 2b). The formation of tau cross-bridges via the N-terminal domain at the interface between microtubules is thus possibly critical to prevent microtubules from collapsing into bundles under macromolecular conditions, as observed by electron microscopy (Fig. 2e).

We have also examined whether tau 2N can dissociate microtubule bundles when they are preformed under macromolecular conditions. As shown in figure S4B, microtubules cannot be released from preformed bundles in the presence of tau 2N. Microtubule bundling under macromolecular crowding can thus be considered irreversible regarding to the role of tau as microtubule spacer. In order to keep microtubules well separated from each other, tau 2N should therefore be located at the interface between microtubules before bundling takes place.

### The N-terminal domain of tau antagonizes microtubule bundling in non-neuronal mammalian cells at low tau:tubulin fluorescence ratio

**Fig. 3:**
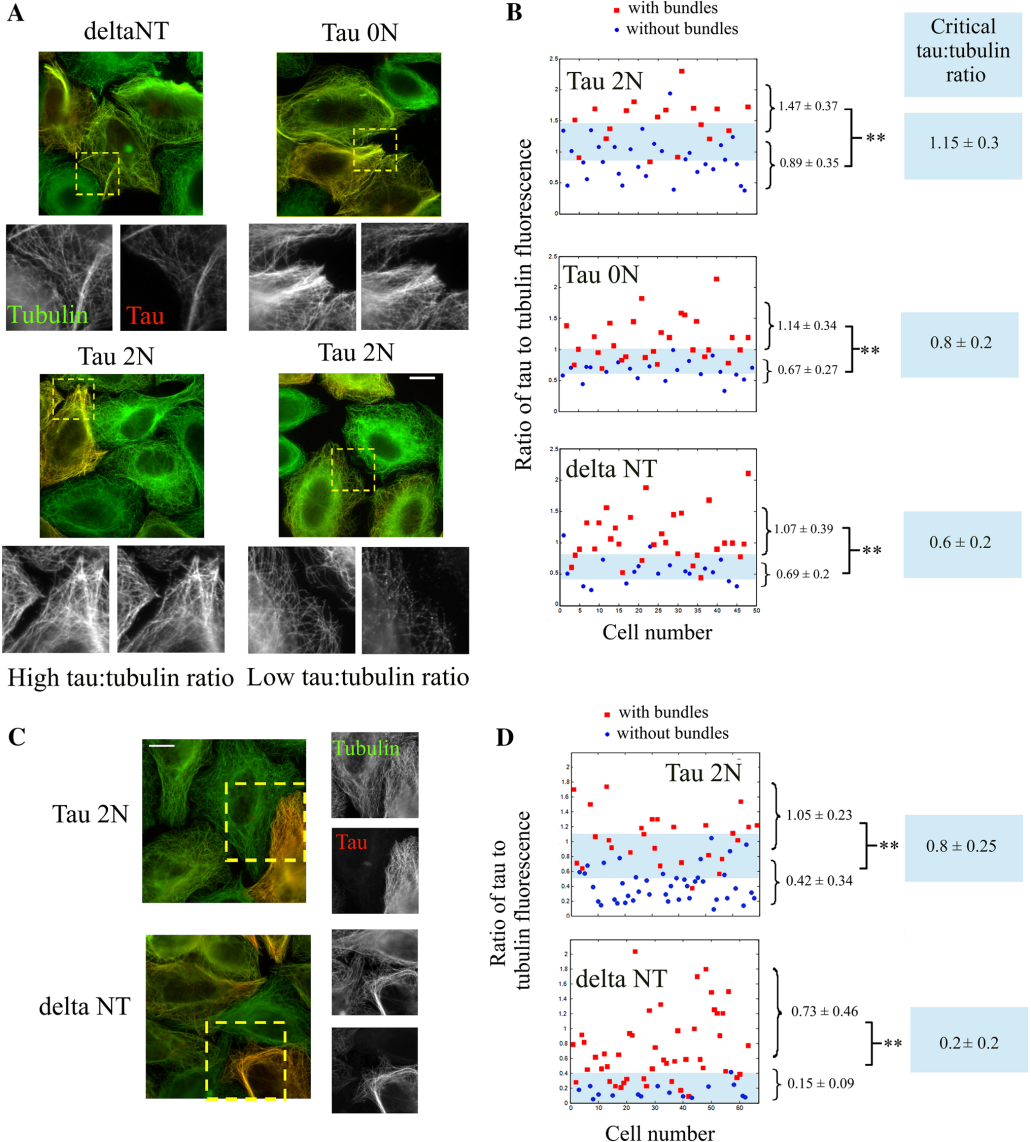
The N-terminal domain of tau antagonizes microtubule bundling in non-neuronal cells at low tau:tubulin fluorescence ratio. **a** Fluorescence microscopy of HeLa cells expressing Tau 2N, Tau 0N and deltaNT, as indicated in the figure. Anti-tubulin and anti-Tau immunofluorescences are represented in *red* and *green* respectively. The anti-tau antibody is directed against the C-terminal domain of tau and recognizes all the tau constructs used in this study. *Scale bar* 30 µm. **b** Statistical analyses of the formation of microtubule bundles in HeLa cells vs tau:tubulin fluorescence ratio for the three tau constructs. For both cells displaying microtubule bundles or not (see supplementary figure S7), the mean fluorescence ratios and standard deviations were determined. For all tau constructs, the ratios of tau:tubulin fluorescence were significantly different in cells with and without microtubule bundles as indicated in the figure. **p < 0.01; two-tailed *t* test. Tau 2N induces microtubule bundling at a significantly higher tau:tubulin fluorescence ratio than deltaNT and to a lesser extent Tau 0N. The transition between bundling and no bundling occurs at a critical tau:tubulin fluorescence ratio indicated in the *figures* for each tau constructs. The critical fluorescence ratio and interval boundaries were estimated as the mean and difference between the ratios leading to 25 and 75 % of cells having microtubule bundles, respectively. The transition zone between bundling and no bundling is represented in *blue* in the figures. **c** Fluorescence microscopy of HeLa cells expressing either tau 2N or deltaNT and treated with 100 nM taxol for 8 h. Microtubule bundling appears as *less marked* in cells expressing tau 2N than in cells expressing deltaNT. *Scale bar* 30 µm. **d** Same as **b** for taxol-treated cells. Again, a higher tau:tubulin fluorescence ratio is required to induce microtubule bundling in cells expressing tau 2N than deltaNT

In contradiction with the experimental results obtained under macromolecular crowding conditions in vitro, tau overexpression in non-neuronal mammalian cells leads to the appearance of microtubule bundles [25, 26, 31, 32] (Fig. 3a). This fact has established tau as a bundling factor. However other facts have nuanced this view: (1) Experimental results showed that the N-terminal domain was not required for microtubule bundling ([7], Fig. 3a). This is surprising as this domain is responsible for microtubule bundling in vitro; (2) Microtubule stabilization by taxol is sufficient to induce the formation of microtubule bundles [52], which indicates that Tau, solely via its microtubule-stabilizing activity, could induce microtubule bundling [7]; (3) When overexpressed in cells, many microtubule partners lead to microtubule bundling while it is not their primary functions. EB-1 is a protein which recognizes specifically the growing plus-ends of microtubules. When artificially overexpressed in cells, EB-1 stabilizes microtubules and leads to the formation of thick bundles [53]. Spastin, a microtubule-severing enzyme, is another example of a protein [54] that forms microtubule bundles when overexpressed and prevented from cutting microtubules in cells.

To better understand the role of tau on the spatial organization of microtubules in a cellular context, we analyzed the formation of microtubule bundles versus the tau:tubulin fluorescence ratio on microtubule structures in HeLa cells expressing tau 2N, tau 0N and deltaNT (see figure S5). The tau:tubulin fluorescence ratio should reflect the tau:tubulin molar ratio on microtubules. This parameter is therefore more informative than the percentage of cells displaying microtubule bundles [7]. At low tau:tubulin fluorescence ratios, neither of the tau constructs induces bundling (Figs. 3a, b and S5). In contrast, at high tau:tubulin fluorescence ratios, all the tau constructs induce bundling. The difference between the three tau constructs lies in the critical tau:tubulin fluorescence ratio required to induce microtubule bundling (Fig. 3b). Microtubule bundling appears at a significantly higher expression level for the longest tau isoform, tau 2N, than for deltaNT and, to a lesser extent, for the shortest isoform of tau, tau 0N. This result indicates that the N-terminal part of tau counteracts microtubule bundling as observed in vitro under macromolecular crowding conditions. To exclude the influence of varying microtubule stability, the same experiments were repeated in HeLa cells treated with taxol to stabilize microtubules and the results again indicate a negative regulation of microtubule bundling by the N-terminal domain of tau at low tau:tubulin fluorescence ratios (Fig. 3c, d).

We also considered microtubule bundling after osmotic shock in taxol-treated HeLa cells. Osmotic stress increases intracellular macromolecular crowding and leads to the formation of microtubule bundles, as previously reported [55]. In line with this, NaCl treatment indeed leads to the formation of microtubule bundles in control HeLa cells (figure S6A and B). Interestingly, the percentage of cells displaying microtubule bundles is not increasing significantly between cells expressing tau 2N under control and hypertonic conditions (figure S6A and B). Again, in cells displaying a low tau:tubulin fluorescence ratio, the presence of microtubule bundles after osmotic stress is less marked than at high tau:tubulin fluorescence ratio (figure S6C). However cells expressing deltaNT formed microtubule bundles whatever they were exposed to osmotic stress or not. Altogether these results indicate that the N-terminal domain of tau 2N, at low tau:tubulin ratio, antagonizes microtubule bundling in a cellular context.

### An estimation of the tau:tubulin molar ratio in axons of mouse cortical neurons reveals that tau may not trigger but rather prevent the formation of microtubule bundles

**Fig. 4:**
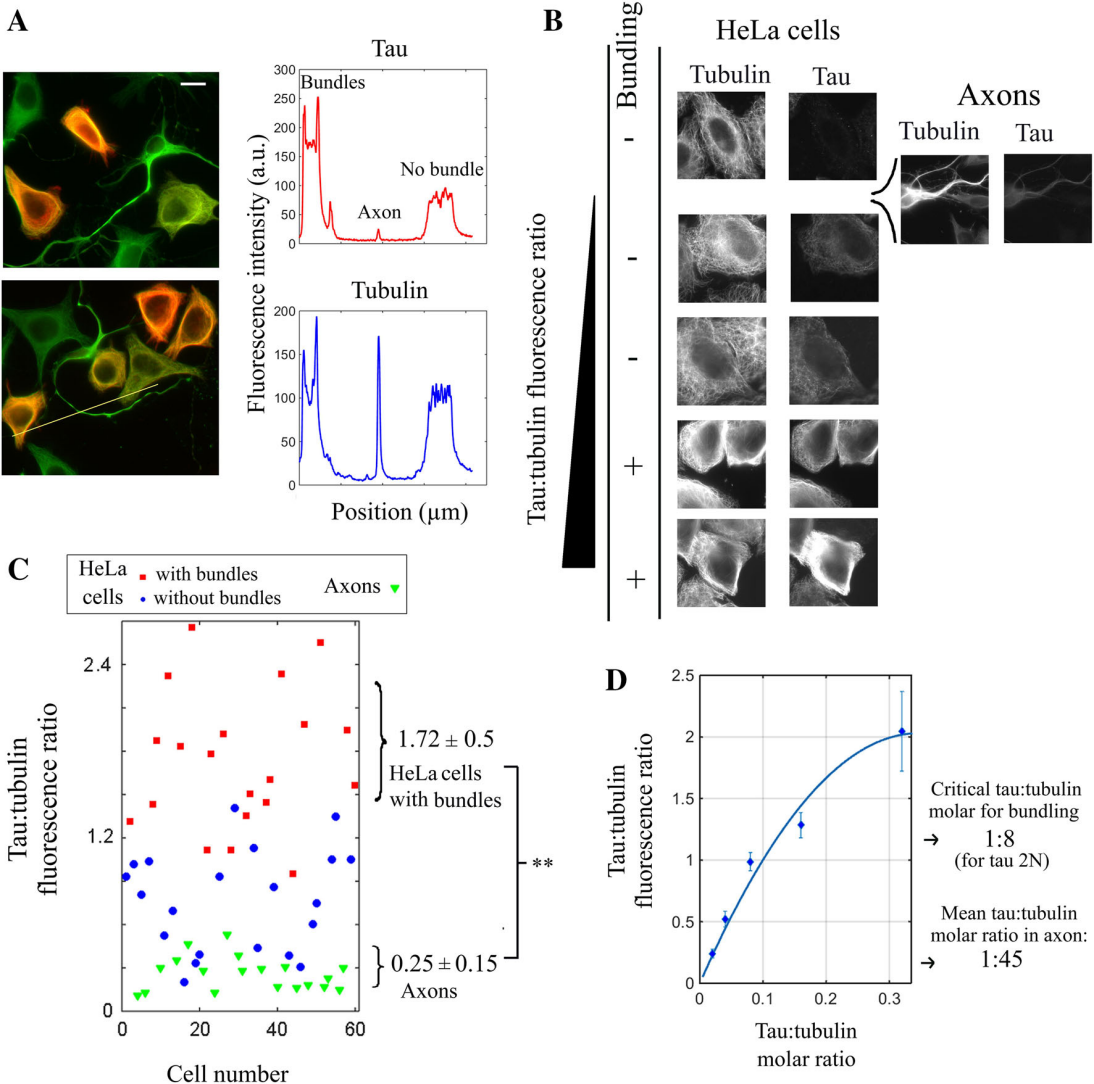
In axons, the tau:tubulin molar ratio is not sufficient to trigger microtubule bundling and should rather prevent microtubules from bundling. **a** *Left panel* primary mouse cortical neurons were cultured for 7 days and then co-cultured with HeLa cells expressing Tau 2N for 8 h. Fluorescence microscopy reveals the expression levels of Tau in neurons and some Hela cells. The anti-tau and anti-tubulin antibody fluorescence is represented in *red* and *green*, respectively. The anti-tau antibody recognizes an epitope in the C-terminus of tau which is conserved in both mouse and human. *Scale bar* 30 µm. *Right panel* fluorescence intensities of anti-tau and anti-tubulin antibodies along the *yellow line* represented in the *bottom* image of the *left panel*. The positions of the cells with or without microtubule bundles and the axon are indicated in the *figure*. **b** Images of HeLa cells and a representative axon sorted by their respective tau:tubulin fluorescence ratio. In axons, the tau:tubulin fluorescence ratio appears far lower than in HeLa cells having microtubule bundling. **c** Statistical analyzes of the tau:tubulin fluorescence ratio measured in axons (*green triangles*) and HeLa cells (*circles* and *squares*) as described in Fig. 3. The axonal tau:tubulin fluorescence ratio is indeed significantly lower than in HeLa cells displaying microtubule bundles (we controlled that the presence of HeLa cells does not change the tau:tubulin fluorescence ratio measured in cultured neurons). **d** Calibration *curve* of the ratio of tau:tubulin fluorescence vs tau:tubulin molar ratio, which was measured from microtubules interacting with recombinant Tau 2N in vitro. Each *dot* represents the average over ten different measurements. The putative tau:tubulin molar ratio in axons was estimated by using the calibration curve and the mean tau:tubulin fluorescence ratio found in axons (0.17). The critical tau:tubulin molar ratio required to form microtubule bundles in HeLa cells was obtained by using the critical tau:tubulin fluorescence ratio for microtubule bundling (1.15 for tau 2N). The calibration curve was fitted with a second order polynome using a least *square* fitting

Tau promotes microtubule bundling in HeLa cells at elevated tau:tubulin fluorescence ratios while the N-terminal domain responsible for tau-mediated cross-bridges clearly antagonizes their formation at lower ratios. We then wondered which of these two regimes prevails in axons of mature primary neurons. To explore this point, we co-cultured primary mouse cortical neurons and HeLa cells expressing tau 2N and measured the tau:tubulin fluorescence ratio in both axons and transfected HeLa cells (Fig. 4a). HeLa cells thus serve as internal controls to gauge the critical tau:tubulin fluorescence ratio for microtubule bundling. We noticed that the tau:tubulin fluorescence ratio was significantly lower in axons than in HeLa cells displaying microtubule bundles (Fig. 4a–c). To make a parallel between in vitro results with those obtained in cells, we recorded a calibration curve of the measured tau:tubulin fluorescence ratio on microtubules deposited on mica versus tau:tubulin molar ratio (Figs. 4d and S2). Using this calibration curve, the estimated value of the mean tau:tubulin molar ratio along axons is about 1:45, which is in line with the values previously reported. By comparison, in order to trigger microtubule bundling in HeLa cells, the tau:tubulin molar ratio should be larger than 1:8 for tau 2N. The tau:tubulin molar ratio in axons of cultured cortical neurons is thus probably not sufficient to induce microtubule bundling. These results are in line with an inhibition of microtubule bundling orchestrated by the N-terminal domain of tau. However, precautions should be taken before generalizing these results. The tau:tubulin of cortical neurons in culture for 7 days may not be representative of all neurons. In addition, the tau:tubulin ratio is not stable along axons and may increase in the apical region [56]. We thus cannot exclude that tau can initiate microtubule bundling in specific locations in axons.

### Both numerical and analytical analyses predict that tau could be an efficient microtubule spacer owing to tau diffusion on microtubules

**Fig. 5:**
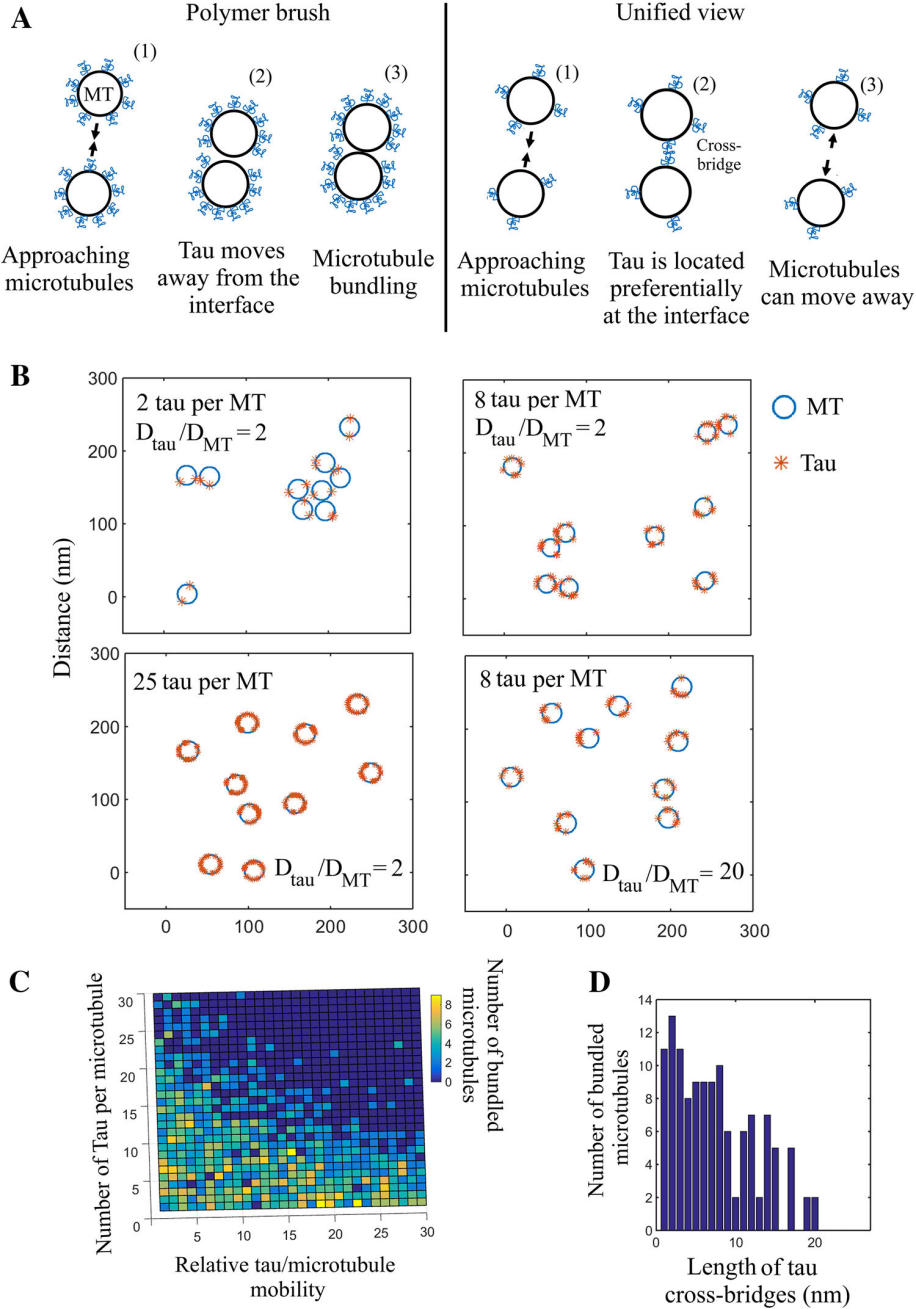
Numerical simulations of the mechanism by which tau acts as a microtubule spacer. **a** Schematic representation of the polymer brush model and the alternative model proposed based on tau cross-bridges and tau diffusion. (1) In the polymer brush model, tau can move away from the interface between microtubules and thus cannot prevent microtubule bundling under macromolecular crowding conditions, unless sterical hindrance prevents tau movements at elevated tau:tubulin molar ratios. (2) Cross-bridging of tau at the interface between microtubule provides an energy benefit to place tau at the interface between microtubule. Tau cross-bridges then keep microtubules at distance and prevent short-range attraction between microtubules under macromolecular crowding conditions. **b** Numerical simulations of the spatial distribution of 10 microtubules moving on a 300 × 300 nm^2^ area. The number of tau molecules per microtubule, *N*, and the relative tau mobility, $\frac{{D_{\text{tau}} }}{{D_{\text{MT}} }},$ are as indicated in the figure. We remarked that both increasing the number of tau proteins per microtubule and the tau diffusion decreases the occurrence of bundling. *r*_p_ = 5 nm (range of excluded volume interactions); *r*_c_ = 15 nm (range of tau cross-bridges). *Number* of iterations: 10^5^. *L* the length of the microtubule, is 500 nm. See supplementary data 1 for details about the model. Here are represented the transverse views of the microtubule array. Tau molecules having different positions along microtubules are then projected on the section view. *D*_tau_,/*D*_MT_ values ranging from 2 to 20 were used for the numerical simulations. This choice is based on the theoretical diffusion constant of a cylindrical molecule (Eq. 1, supplemental data 1), the estimated value of the cytoplasm viscosity and the varying length of microtubules (see supplemental data 1 for details). However, as *D*_MT_ in axons has not been measured, these values may be considered as arbitrary. **c** Number of bundled microtubules after completion of the numerical simulations vs the number of tau proteins per microtubule and the relative tau mobility along microtubules (*D*_tau_/*D*_MT_). Same conditions as **b**. **d** Numerical simulations indicating the number of bundled microtubules among 20 microtubules moving on a 350 × 350 nm^2^ area vs the range of tau cross-bridges, *r*_c_. Number of iterations: 10^5^. *D*_tau_/*D*_MT_ = 3. The other parameters are the same as B)

In the polymer brush model [57], tau is an unstructured polymer which coats the surface of particles to prevent their aggregation. For an efficient steric hindrance in the case of microtubules, scaling laws indicate that about 1 tau molecule for 6 tubulin dimers is required [40] (see Eq. 6 in supplementary data 1). The microtubule surface should then be half-saturated with tau (saturation ratio: 1:3), which is much too high to be relevant under physiological conditions. Along with this, tau diffuses on microtubules. If there is no force to keep tau at the interface between approaching microtubules, tau has the possibility to move away from the interface of microtubules via thermal diffusion (Fig. 5a). Consequently, tau cannot efficiently block microtubule bundling at low tau:tubulin ratios in the polymer brush model.

In the cross-bridge model, tau dimerization via the N-terminal domain can provide an energy benefit to keep tau at the interface between microtubules and prevent tau from bundling under macromolecular crowding conditions (Fig. 5a). In addition, tau cross-bridges will oppose the further approach of two interacting microtubules. The compression of tau cross-bridges at the interface between approaching microtubules indeed generates a repulsive force [58] and, accordingly, tau cross-bridges have been considered as strings [59]. To perform numerical simulations, we considered that tau forms antiparallel cross-bridges at the interface between microtubules and assumed that tau dimerization can be disrupted when interacting microtubules move away from each other. The latter assumption seems justified as a massive microtubule bundling was not observed at low tau:tubulin ratios (Fig. 2). Based on these hypotheses, the results of numerical simulations indicate that tau 2N is an efficient spacer for microtubules (Fig. 5b, Videos 1–4). Interestingly, tau diffusion reduces the critical tau:tubulin ratio required to prevent microtubule bundling. As the microtubule surface area scanned by tau increases with tau mobility, less tau is required to keep microtubule separated (Fig. 5b, c). To emphasize this point, an analytical approach shows that the number of diffusing tau proteins required to prevent the formation of microtubule bundles decreases linearly with the relative mobility of tau, $D_{\text{tau}} ,/D_{\text{MT}}$ where *D*_tau_, *D*_MT_ are the diffusion constants of tau and microtubules, respectively (see supplementary text, Eq. 5). Interestingly, increasing the length of the N-terminal domain significantly reduces the number of tau molecules required for keeping microtubule separated (Fig. 5d and Eq. 5 in supplementary data 1).

## Discussion

**Fig. 6:**
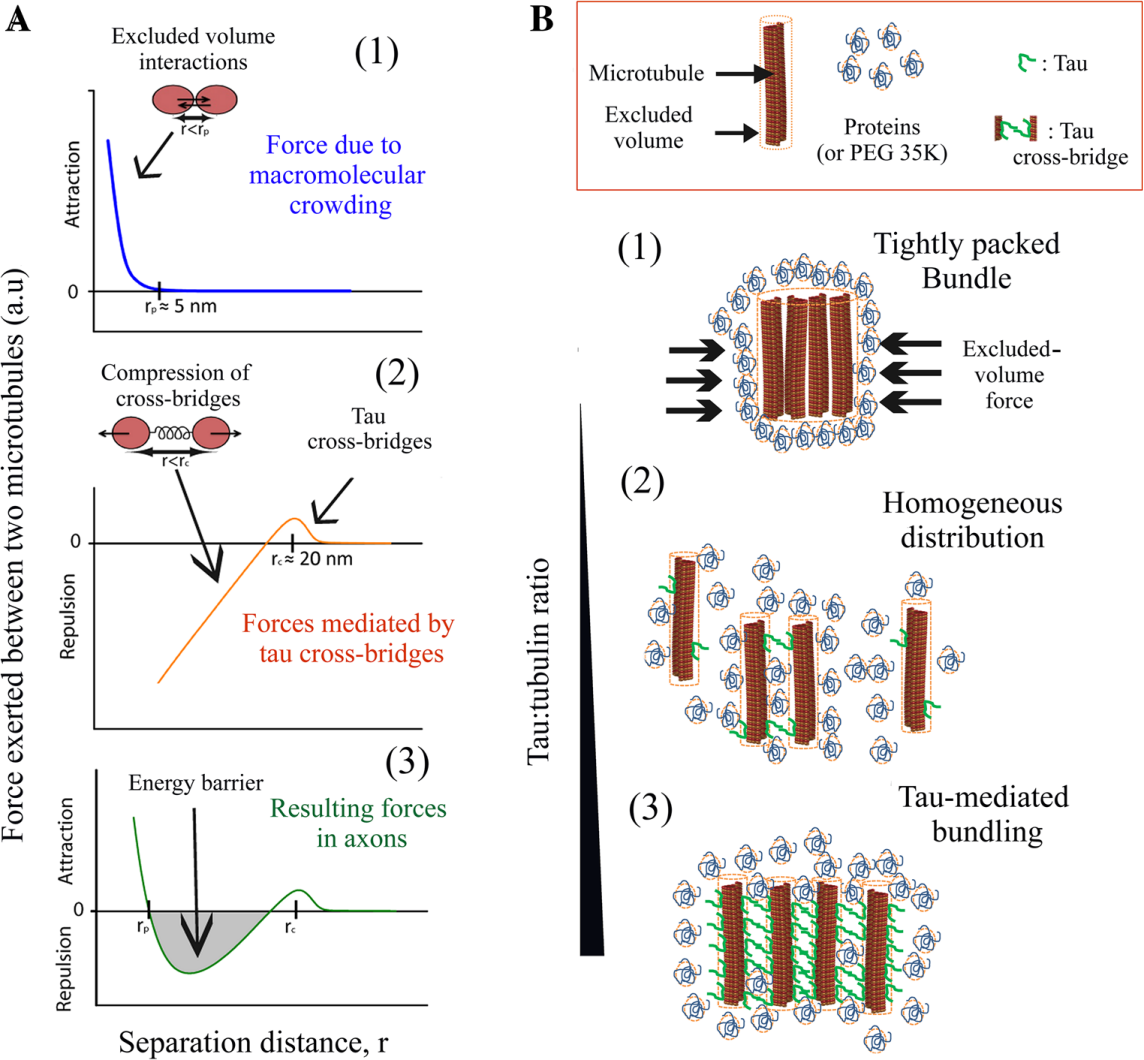
Spatial organization of microtubules in the presence of tau under macromolecular crowding conditions. **a** Schematic curves representing forces vs separation distance between microtubules. *1* Macromolecular crowding induces a strong short-ranged attraction (*r* < 5 nm, average size of proteins) and the formation of tightly packed microtubule bundles. *2* The formation of tau cross-bridges between microtubules generates a long-ranged attraction (about 20 nm). However the compression of tau cross-bridges generates a repulsion force at shorter distances. *3* By combining the forces due to macromolecular crowding and tau cross-bridges, we notice the appearance of an energy barrier which could prevent microtubules from collapsing into tight bundles in axons. **b** Spatial organization of microtubules at various tau:tubulin molar ratios. *1* In the absence of tau or at very low tau:tubulin molar ratios (<1:60 for tau 2N, in vitro), macromolecular crowding induces the collapse of microtubules into tight bundles. *2* At moderate tau:tubulin molar ratios (from about 1:60 to 1:15, in vitro), transient tau cross-bridges keep microtubules separated and prevent the formation of tightly packed microtubule bundles. No massive bundling is observed. We propose that this regime prevails in axons. *3* At elevated tau:tubulin ratios (>1:15), the formation of many tau cross-bridges induces the assembly of microtubule bundles in which the spacing between microtubules is about 15–20 nm. This regime prevails when tau is strongly over-expressed in HeLa cells

While microtubules form bundles in non-neuronal cells overexpressing tau, microtubules appear as rather homogeneously distributed in axonal sections of mature neurons [16, 17, 19]. The spatial separation of microtubules most probably favors long-range transport of vesicles, mitochondria and RNA along axons. To explain the spatial separation of axonal microtubules, tau was then considered as a microtubule spacer [21, 22]. In the polymer brush model, the separation between microtubules is due to the unstructured N-terminal domain of tau which acts as a repulsive layer. While such a mechanism deserves to be considered, its application to tau and axonal microtubules has to be carefully analyzed. The point is that near-saturating concentrations of tau are required to form a continuous repulsive layer on microtubule surface. In axons, the tau:tubulin molar ratio ranges between 1:12 and 1:68 [41–43] and, here, an estimation based on calibrated immunofluorescence in axons of cortical neurons leads to a tau:tubulin molar ratio of about 1:45. The tau:tubulin molar ratios found in axons are therefore not sufficient to form a continuous layer on microtubule surface. Importantly, tau diffuses on the microtubule lattice. Tau has thus the possibility to move away from the interface between microtubules. In the absence of additional factors, microtubules should then form bundles under macromolecule crowding conditions (Figs. 5a, 6a).

To understand the mechanisms responsible for separating axonal microtubules, we considered that tau forms cross-bridges at the interface between microtubules. The energy benefit of forming cross-bridges allows the specific location of tau at the microtubule interface and thus prevents tau from moving away (Fig. 1a). At low tau:tubulin molar ratios, the formation of only few cross-bridges is not sufficient to trigger the formation of microtubule bundles by itself. However, when microtubules further approach from each other, the compression of tau cross-bridges generates an energy barrier. Consequently, the short-range attraction force due to macromolecular crowding can no longer take place (Fig. 6a), which precludes the formation of tightly packed microtubule bundles. In cells over-expressing tau, tau-cross-bridges are numerous and artificially induce microtubule bundling (Fig. 6b). In vitro, only elevated tau:tubulin molar ratios and moderate ionic strengths allow the clear detection of microtubule bundles (Figs. 1 and S3). The biphasic effect of tau on the spatial organization of microtubules is misleading and led to the biased view that tau promotes microtubule bundling in vivo.

Interestingly, thermally-induced movements of tau allow the exploration of a large surface area on the microtubule lattice in search for other tau proteins on the surface of other microtubules. Tau diffusion is thus critical to prevent microtubule bundling at tau:tubulin molar ratios well below the ratio required to saturate microtubules (Figs. 5b, c, 6b).

The unified view on the role of tau in axonal microtubule organization presented in this study provides new insights into the role of tau in neuronal functions. For example, tau was reported to impair the long-range transport of vesicles and mitochondria along microtubules via molecular motors, as observed repeatedly in cells overexpressing tau [60–63]. However, both tau diffusion on microtubules and its ability to form cross-bridges in between microtubules enable to keep microtubules separated at low tau levels. At such low levels, the presence of tau on microtubules should therefore not constitute an obstacle for active transport of cargoes.

The consequences of alternative splicing of tau mRNA and tau phosphorylation on the spatial distribution of axonal microtubules also deserve to be considered and could enlighten the process leading to axon degeneration observed in Alzheimer’s disease. The results present here show that the tau isoform with the longest N-terminal domain (2N) is the most potent to prevent microtubules from bundling under macromolecular crowding conditions. During neurogenesis, tau isoforms with short N-terminal domain (0N and 1N) are expressed but their expression is significantly reduced in adult brain [64]. In mature axons, the presence of tau 2N could thus be necessary to keep microtubules homogeneously distributed. The role of the number of microtubule binding repeats (4R or 3R) may also matter. 4R isoforms bind to microtubules with a higher affinity than 3R isoforms but a strong binding also limits tau diffusion on microtubules. Experimental data are thus required to clarify whether tau 4R or 3R have a similar ability to prevent microtubule bundling.

In addition, tau phosphorylation leads to a lower affinity of tau for microtubules [65], which is considered critical for the redistribution of tau to the somatodendritic compartment and the accumulation of tau aggregates [66]. Our unified model however provides an alternative view. Tau phosphorylation events and especially those occurring in the proline rich and N-terminal domain may alter the capacity of tau to separate microtubules. An early event in Alzheimer’s disease could thus be the collapse of axonal microtubules into tightly packed bundles due to inappropriate tau phosphorylation events. Further investigations should be carried out to explore this hypothesis.

## Materials and methods

### Preparation of sheep brain tubulin and subtilisin-treated tubulin

Tubulin was purified from sheep brains and stored at −80 °C in 20 mM MES–KOH, pH 6.9, 0.5 mM DTT, 0.5 mM EGTA, 0.25 mM MgCl_2_, 3.4 M glycerol, and 0.1 mM GTP. Before use, an additional cycle of polymerization was performed, and tubulin was resuspended in 20 mM MES–KOH, pH 6.9, 0.25 mM EGTA, 0.25 mM MgCl_2_. Tubulin concentration was determined by spectrophotometry using an extinction coefficient _*ϵ*278 nm_ = 1.2 × 10^5^ M^−1^ cm^−1^ [28].

### Turbidimetry measurements

The kinetics of microtubule assembly and (or) bundling was monitored by turbidimetry at 370 nm using an Ultrospec 3000 spectrophotometer (GE Healthcare, Fairfield, CT) equipped with a temperature controller. Microtubule assembly was obtained after preincubating tubulin samples on ice for 5 min in polymerization buffer (10 mM HEPES–KOH pH 6.8, 30 mM KCl, 20 % glycerol, 1 mM EGTA, 4 mM MgCl_2_, 1 mM GTP) in the presence or absence of tau. Tubulin polymerization was then initiated by shifting the temperature to 37 °C. Microtubule bundling was monitored by turbidimetry at 37 °C after adding either 1 % PEG 35K or tau at varying concentrations to taxol-stabilized microtubules in the indicated buffer.

### Cloning of tau isoforms

Tau 2N/4R and deltaNT cDNAs were obtained by amplifying hTau40 pET29b plasmid (catalogue no. 16316, Addgene, Cambridge, MA) either from the beginning or from P172 to L441 using Phusion Hot Start II High-Fidelity DNA Polymerase (catalogue: F-537L, Thermo Fischer Scientific). The PCR products were first cloned into pENTR/D-TOPO by TOPO cloning (primers are listed in Supplemental Table 1) and then moved into pDEST17 by the LR reaction for recombinant protein expression with a His-Tag (Life Technologies). To obtain hTau24 three PCRs was necessary) using Hot Start II High-Fidelity DNA Polymerase (catalogue: F-537L, Thermo Fischer Scientific). Tau full length plasmid was used as template to design the primers and to amplify two fragments encoding hTau24 sequence (accession number NM_016834.4). For all PCRs, cycling was a 30 s initial denaturation at 98 °C, 35 cycles with 30 s denaturation at 98 °C and a 30 s annealing at 72 °C and a final extension also at 72 °C for 5 min. PCRs product was cloned into pENTR/D-TOPO by TOPO cloning then moved into pDEST17 by the LR reaction.

### Production of Human Tau Protein

Rosetta-gami™ 2 competent cells were transformed with hTau40 (largest isoform), hTau24 (shortest 4R isoform), and deltaNT (mutant) expressed in Gateway^®^ pDEST™17 Vector (catalogue no. 11803-012, Life technologies). Bacteria were grown in LB medium in the presence of 100 μg/ml ampicillin and 15 µg/ml Chloramphenicol. Overexpression was induced at *A*_600 nm_ = 0.5 with 0.7 mM isopropyl *β*-d-thiogalactopyranoside, and incubation was continued for 3.5 h at 37 °C. Bacteria were pelleted by a 10-min, 4000×*g* centrifugation, and the pellet was resuspended in Buffer A (25 mM Tris–HCl, 25 mM Mes–KOH, 500 mM NaCl, 0.2 mM MgCl_2_, 1 mM PMSF, 5 mM DTT, pH 7.5). Bacteria were then disrupted by sonication, and centrifuged for 10 min (4000×*g*), the supernatant was then boiled for 20 min, and ultracentrifuged at 100,000×*g* for 45 min at 4 °C. Clarified cell lysate was then loaded on a Ni^2+^ -nitrilotriacetic acid column (HisTrap HP, 1 × 1 ml, GE Healthcare Life Sciences). The proteins were eluted with 5 column volumes of buffer B (25 mM Tris–HCl, 25 mM Mes–KOH, 250 mM NaCl, 250 mM Imidazole, 0.2 mM MgCl_2_, 1 mM PMSF, 5 mM DTT, pH 7.5). Purified proteins were then dialyzed overnight at 4 °C to cleave the His-Tag against 20 mM Tris, 20 mM Mes, 250 mM NaCl, 0.5 mM EDTA, 1 mM DTT, pH 7.5) using TEV protease (catalogue no. T4455, SIGMA). This step was followed by a second nickel-affinity chromatography to remove the uncleaved recombinant tau and TEV protease. Fractions of interest were combined and dialyzed against 25 mM Hepes, 250 mM NaCl, 0.25 mM DTT, 0.1 mM PMSF, pH 7.4 to eliminate traces of imidazole. Proteins were concentrated by ultrafiltration (Corning^®^ Spin-X^®^ UF 500, 10 kDa cut-off) and the final concentration of Tau was determined by amino acid analysis. All of the purification steps were performed in the presence of complete Protease Inhibitor Cocktail tablets (catalogue no. 00000001187358000, Roche Applied Science).

### Atomic force microscopy

Samples containing microtubules and tau under specified conditions were deposited on freshly cleaved mica and dried for atomic force microscopy (AFM) imaging, using a protocol that we developed [67]. The electrostatic adsorption of microtubules on mica is mediated by magnesium ions present in the buffer. All AFM experiments were performed in peak force mode with Nanoscope V (Bruker/Veeco, Santa Barbara, CA). The peak force tapping mode was performed using silicon tips (Scanasyst-Air-HR, Bruker). The applied force was minimized as much as possible.

### Transmission electron microscopy (TEM)

For ultrathin sectioning, microtubules were prepared with 20 μM tubulin with or without 7 µM of tau and (or) 1 % PEG 35K in 10 mM HEPES–KOH, 50 mM KCl, pH 6.8, 1 mM EGTA, 4 mM MgCl_2_ and 1 mM GTP, 20 % glycerol. Microtubules were pelleted at 20,000×*g* for 30 min at 37 °C. The pellets were gently resuspended in 10 mM HEPES–KOH, 50 mM KCl, pH 6.8, 1 mM EGTA, 4 mM MgCl_2_ and 1 mM GTP, 20 % glycerol, 1 % glutaraldehyde, 0.2 % tannic acid and incubated for fixation for 1 h at room temperature. Samples were then post-fixed with 1 % osmium tetroxyde in cacodylate buffer 0.1 M, pH 7.3. After dehydration in ethanol bathes of increasing concentrations, pellets were embedded in epoxy resin (Embed-812 Embedding kit #14120, EMS). Collodion-carbon-coated copper grids were used to collect the ultrathin sections of 40 nm thickness. The sections were then stained sequentially with 2 % uranyl acetate aqueous and Reynold’s solutions and analyzed in bright field mode using a Zeiss 902 transmission electron microscope. Images were acquired using a Megaview III CCD camera with the iTEM software (Olympus Soft Imaging Solution) at a magnification of 79,000×.

### Immunofluorescence

HeLa cells and (or) neurons were grown on poly-l-lysine-coated glass coverslips and fixed in ice-cold methanol immediately followed by fixation in 4 % paraformaldehyde at 37 °C in PBS for 30 min. After fixation, cells were then washed and incubated for 1 h with mouse monoclonal anti-tubulin antibody (E7, 1:2000 dilutions) and anti-tau antibody (SC-1995, Santa-Cruz, CA). Cells were washed extensively in PBS and incubated for 1 h with fluorochrome (Alexa Fluor^®^ 488 and -555)-coupled secondary antibodies (Invitrogen) in blocking solution. The protocol used to measure the tau:tubulin fluorescence ratios on microtubule structures is described in figure S5.

In vitro, microtubules were polymerized as described in “Turbidimetry measurements” and deposited on freshly cleaved mica. Samples were fixed with 4 % paraformaldehyde in PBS at 37 °C for 10 min. After fixation, samples were then washed three times in PBS and prepared for immunofluorescence as described above. The tau:tubulin fluorescence ratios were measured as described in figure S2.

### Co-culture of neurons and HeLa cells

Cortical neurons from embryonic mice (E19) were prepared as described previously [68] and grown on Poly-l-ornithine-coated (Sigma-Aldrich) 14 mm coverslips at a density of 100,000 cells/coverslips in Neurobasal media, supplemented with 2 % B27, 2 mM l-glutamine. Hela cells previously transfected with 2N tau by using Lipofectamine 2000 (Invitrogen) were co-cultured on neurons at 7-day in vitro (DIV) for 8 h.

### Electronic supplementary material

Below is the link to the electronic supplementary material.

Supplementary material 1 (PDF 772 kb)

Supplementary material 2 (PDF 72 kb)

Supplementary material 3 (WMV 975 kb)

Supplementary material 4 (WMV 981 kb)

Supplementary material 5 (WMV 977 kb)

Supplementary material 6 (WMV 994 kb)
