# Supplementary material for: Role of tau in the spatial organization of axonal microtubules: keeping parallel microtubules evenly distributed despite macromolecular crowding
Source: Cell Mol Life Sci. 2016 Apr 13;73(19):3745–60. doi: 10.1007/s00018-016-2216-z (PMC5002045; doi:10.1007/s00018-016-2216-z)
Supplement: Supplementary file 1 — Supplementary material 1 (PDF 772 kb) [file 18_2016_2216_MOESM1_ESM.pdf]

SUPPLEMENTARY FIGURES S1 TO S7 AND LEGEND TO VIDEOS:

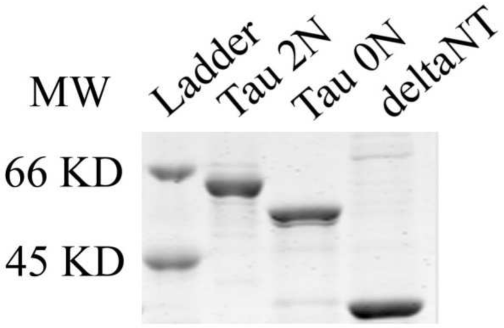

Figure S1: Purified recombinant tau proteins used in this study, as observed after their migration in a denaturating gel.

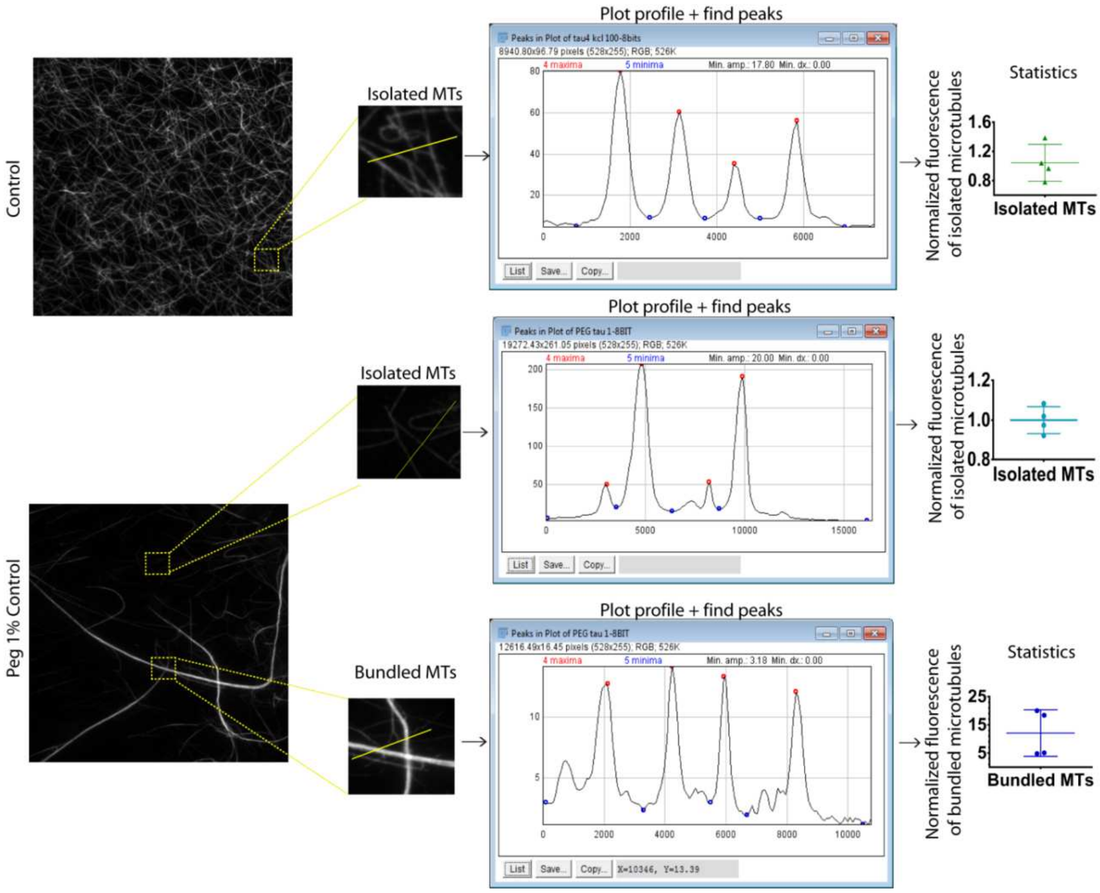

Figure S2: Detection of microtubule bundling from optical images of microtubules deposited on mica.

Microtubules were deposited on mica under indicated conditions and fixed with 4% glutaraldehyde at 37°C. Samples were then prepared for immunofluorescence as for fixed cells. Anti-tubulin antibody was used to detect microtubules. The mean amplitude of the peaks related to the fluorescence of isolated microtubules was determined as described. The amplitudes of the peaks related to microtubule structures were then normalized with this value. Large microtubule bundles have normalized peak intensities higher than that of isolated microtubules. When indicated, the same procedure was used to measure the mean amplitude of the peaks related to the tau fluorescence intensity using anti-tau antibody. The tau:tubulin fluorescence ratio is then the ratio of the mean amplitudes related to tau and tubulin intensities as described in figure S5.
The use of optical microscopy allows to observe microtubule bundling on large area. However, owing to the resolution limit of optical microscopy (about 200-300 nm), microtubules may appear as bundled while in fact they can be isolated with a separation distance shorter than the resolution limit. To discard this hypothesis, electron and atomic force microcopies, which have a lateral resolution in the nanometer range, were used as complementary techniques (figure 1 and 2).

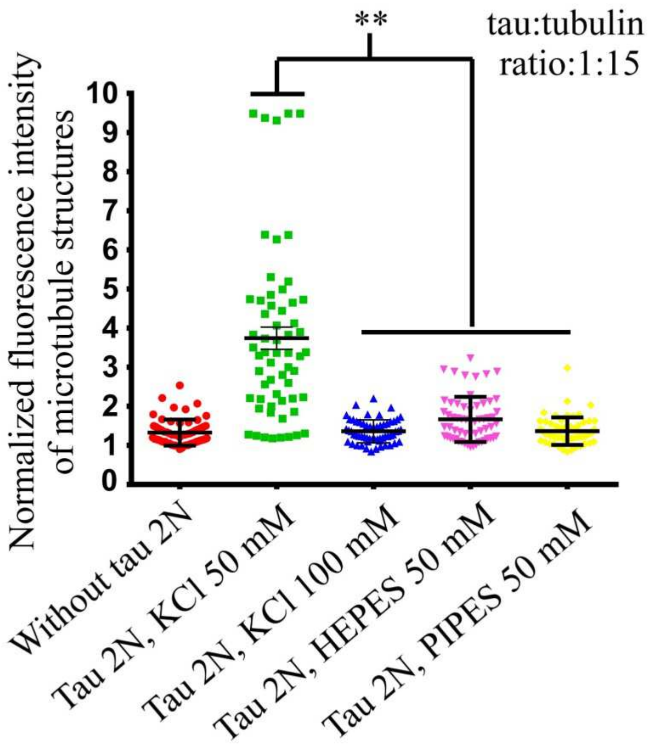

Figure S3: Elevated ionic strength or concentration of zwitterions, HEPES or PIPES, impair microtubule bundling induced by tau2N.

Statistical analyzes of taxol-stabilized microtubules deposited on mica in the presence or absence of tau 2N revealed in varying buffers. 10 mM HEPES-KOH pH 6.8, 30 mM KCl unless stated otherwise, 20% glycerol, 1 mM EGTA, 4 mM MgCl2, 1 mM GTP. HEPES and PIPES pH 6.8 were added at indicated concentration. Results are mean ± SD (n=60). See figure S2 for the description of the method.

A)

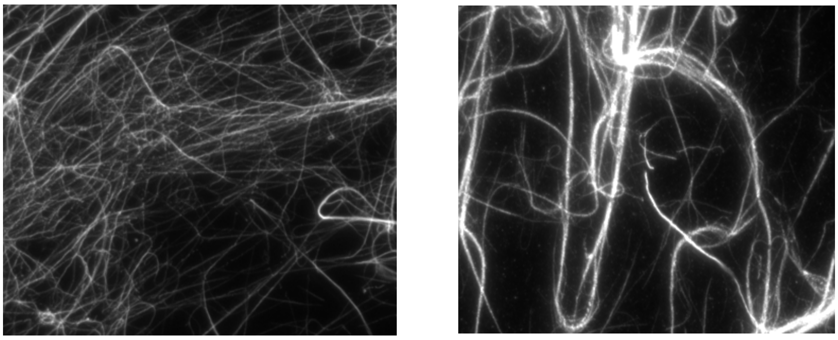

TAU 2N +PEG 35K

KCl 50 mM

TAU 2N +PEG 35K

KCL 100 mM

B)

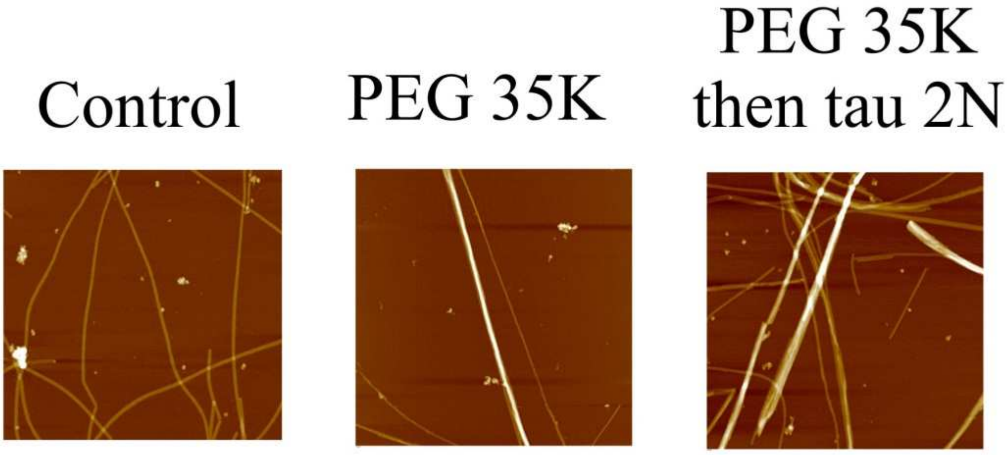

Figure S4: Tau 2N no longer prevent microtubule bundling at high ionic strength and could not dissociate preformed microtubule bundles.

A) Taxol-stabilized microtubules were incubated in the presence of tau 2N (1:15 tau:tubulin ratio) and then exposed to 1% PEG 35K in the presence of 50 or 100 mM KCl. Interestingly, at elevated ionic strength, we noticed the presence of large microtubule bundles while, at lower

ionic strength, tau 2N antagonizes microtubules bundling under macromolecular crowding conditions.

B) Microtubule bundles formed under macromolecular crowding conditions cannot be dissociated by tau 2N afterward.

Atomic force microscopy imaging of microtubules adsorbed on mica in the presence or absence of PEG 35K 1% for 15 min, as indicated. In one of the sample, tau 2N was added after microtubule bundling under macromolecular crowding. Tau 2N failed to dissociate previously formed microtubule bundles. Buffer: 10 mM MES-KOH pH 6.8, 1 mM EGTA, 4 mM $MgCl_2$, 1 mM GTP and 30 mM KCl. Tau:tubulin molar ratio: 1:15.

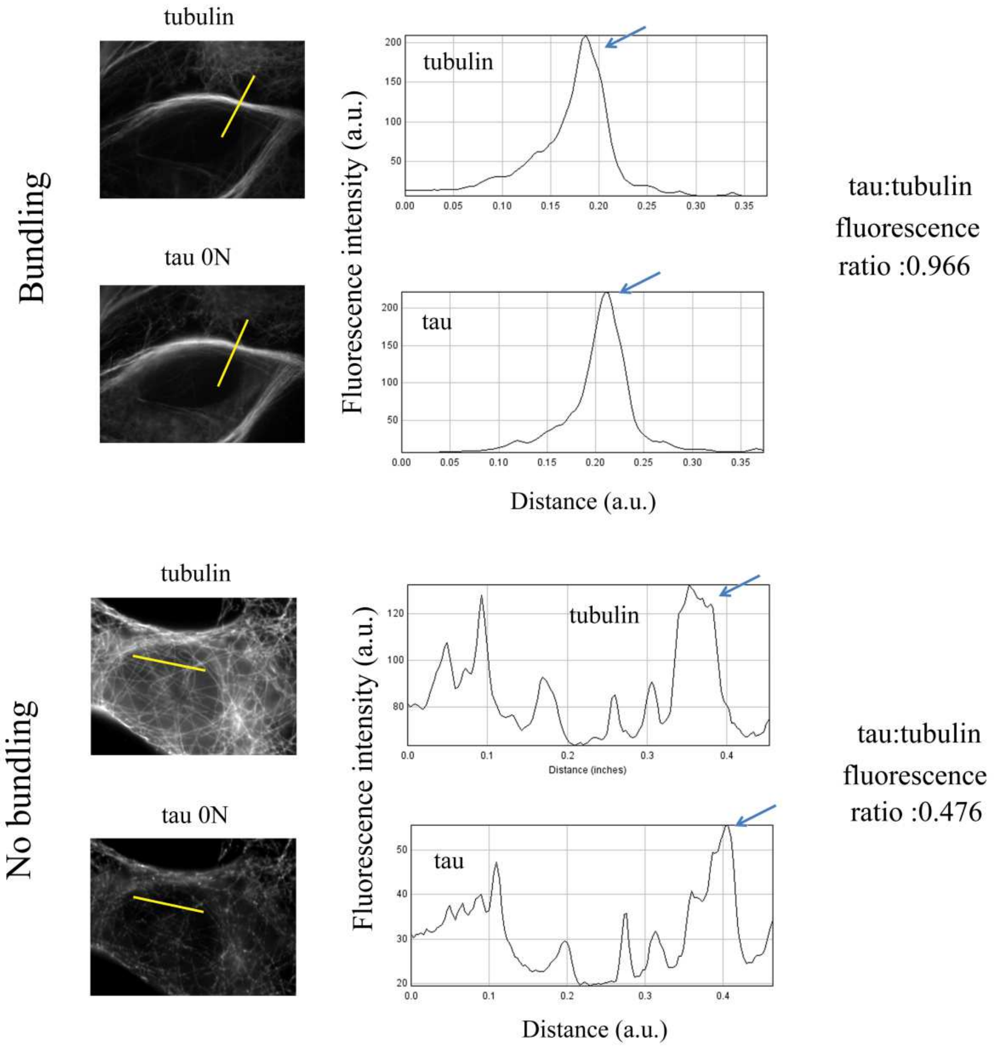

Figure S5: Determination of the tau:tubulin fluorescence ratio in cells

HeLa cells expressing tau constructs at varying level (here tau 0N) were fixed and stained with anti-tau and anti-tubulin antibodies, as indicated. We then recorded the profile of the fluorescence intensity along a line crossing microtubule structures. The peaks of fluorescence intensity relative to tubulin and tau were subtracted by their relative background. The two resulting intensities were then used to determine the tau:tubulin fluorescence ratio. Three ratios were determined on different microtubule structures for each cell and the mean value was reported in figure 3A and D.

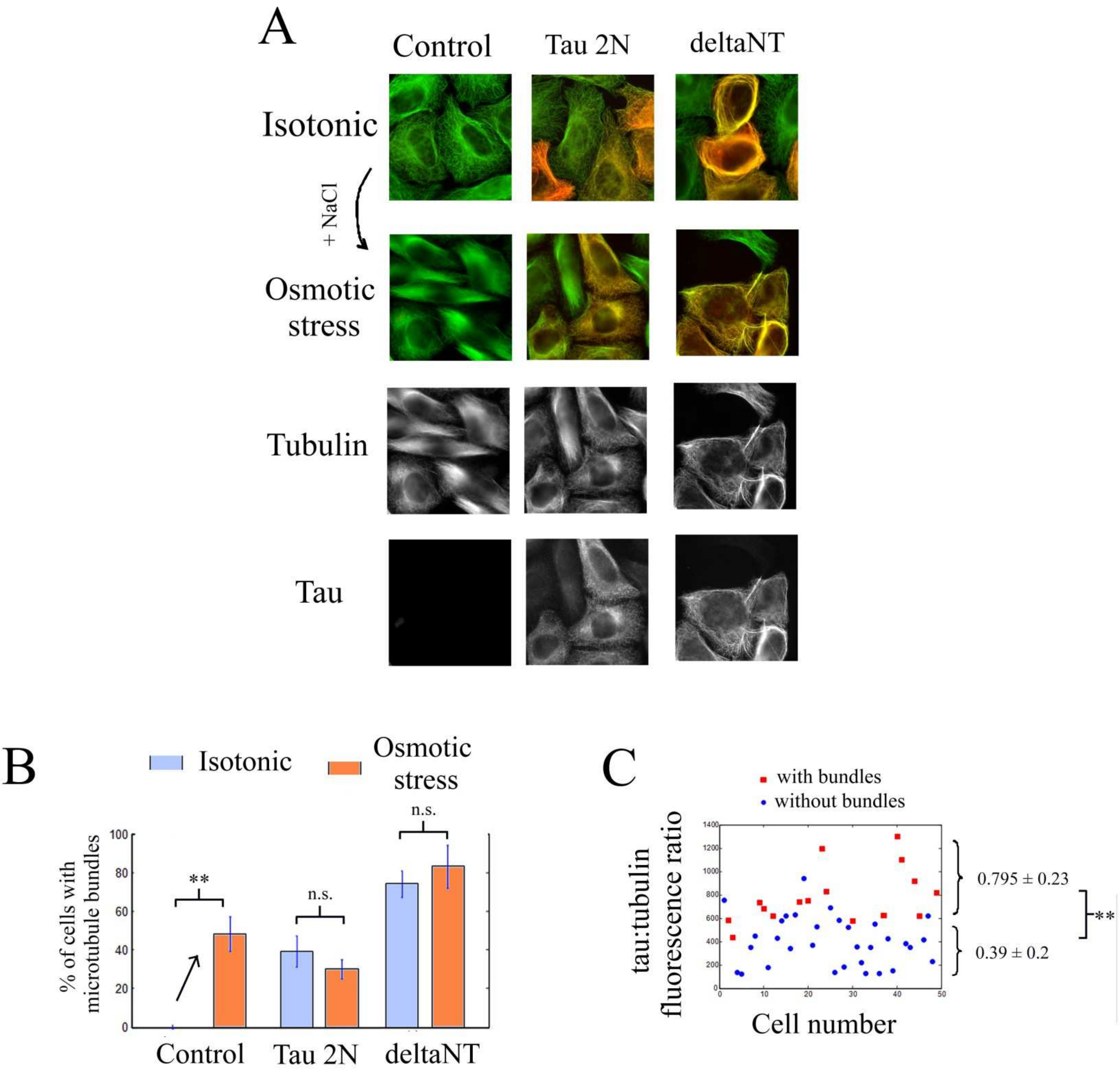

Figure S6: The N-terminal domain of tau limits microtubule bundling under hypertonic conditions in HeLa cells.

A) Upper panel: Immunofluorescence images of taxol-treated HeLa cells expressing either tau 2N or deltaNT after exposure to hypertonic conditions (+ 150 mM NaCl for 6 h). Anti-tau and anti-tubulin immunofluorescences are represented in red and green respectively. We remarked the appearance of microtubule bundles in control cells due to

the hypertonic treatment. In cells expressing tau 2N at low levels, hypertonicity does not further increase the occurrence of bundling.

B) Lower panel: statistical analyzes of the formation of microtubule bundles in HeLa cells after the hypertonic shock. Hypertonicity leads to the formation of bundles in more than 40 % of control cells. In tau 2N-expressing cells, microtubule bundling is not further increase after the hypertonic treatment.

C) Statistical analyzes of the formation of microtubule bundle in HeLa cells expressing Tau 2N at varying tau:tubulin fluorescence ratio. Interestingly, after hypertonic treatment, the occurrence of microtubule bundling is not elevated in cells expressing tau 2N at low tau:tubulin fluorescence ratios.

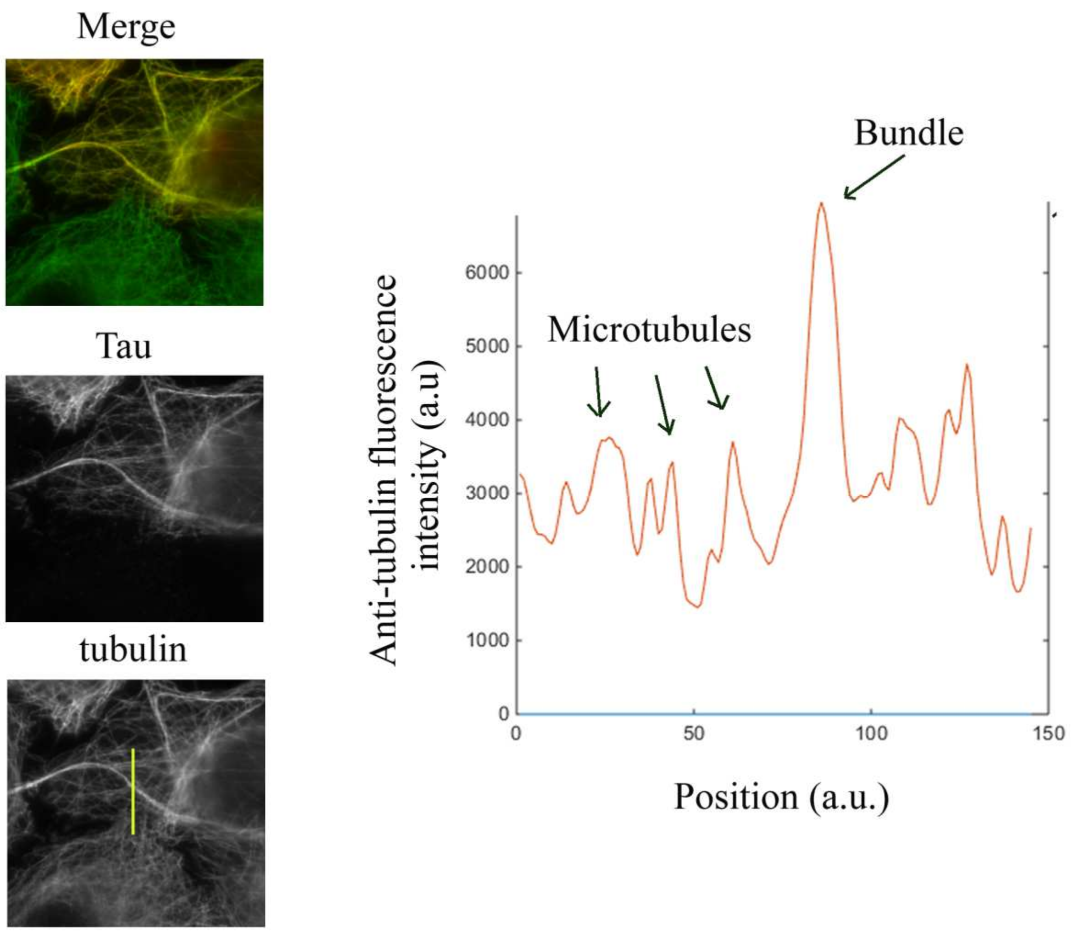

Figure S7: Detection of microtubule bundles in HeLa cells expressing tau (here deltaNT).

A line profile of the anti-tubulin fluorescence was performed to reveal the putative presence of microtubule bundles. The peak heights of the microtubule structures was then measured and compared to average value of the peak of isolated microtubules (the background value was subtracted).When the heights of the peak related to the putative microtubule bundles exceeds by 60% the average peak height of isolated microtubules, we considered that the cell has formed microtubule bundles. In the case represented above, the peak height related to isolated microtubules are in average about 1800 a.u. while that of the putative microtubule bundles is higher than 4000 a.u. We thus considered that this cell has formed microtubule bundles.

LEGENDS TO VIDEOS 1 to 4:

Numerical simulations of the spatial distribution of 10 microtubules moving on a 300×300 nm^2 sectional area. The number of tau molecules and the relative tau mobility, $\frac{D_{tau}}{D_{MT}}$, are indicated in videos. To have clear view of the bundling events, only the transverse section of the parallel microtubules is represented. This is why tau proteins may appear artificially superposed on microtubules.
Number of iterations: 10^5. L, the length of the microtubule, is 500 nm. Videos were recorded by stacking 10^3 images every 10^2 iterations.
