# Supplementary material for: Role of tau in the spatial organization of axonal microtubules: keeping parallel microtubules evenly distributed despite macromolecular crowding
Source: Cell Mol Life Sci. 2016 Apr 13;73(19):3745–60. doi: 10.1007/s00018-016-2216-z (PMC5002045; doi:10.1007/s00018-016-2216-z)
Supplement: Supplementary file 2 — Supplementary material 2 (PDF 72 kb) [file 18_2016_2216_MOESM2_ESM.pdf]

Supplementary information:

Description of the model which describes how tau may separate microtubules:

We first consider that microtubules are aligned and organized in arrays as in axons. Each microtubule in these arrays can undergo thermal diffusion in the direction perpendicular to its axis. The constant of diffusion is (Hamon et al., 2011; Yu and Carlsson, 2004):

$$D_{MT} = \frac{K_B T ln\left(\frac{L}{2R}\right)}{4\pi\eta L} \qquad (1)$$

where T is the temperature; K_B the Boltzmann constant; L and R, the length and radius of microtubules; η, the viscosity of the axoplasm.

We consider that two adjacent microtubules may approach from each other via thermal diffusion. When the separation distance between the two microtubules is shorter than r_p, the range of the excluded volume interactions, microtubules are irreversibly attracted and bundling occurs. Such scenario happens in the absence of tau.

However, in the presence of tau, tau cross-bridges which can be formed at the interface between approaching microtubules can prevent microtubules from bundling. The range of tau cross-bridges, r_c (about 20 nm) is indeed significantly larger that the range of excluded volume interactions, r_p (about 5 nm). During the time that the separation distance between two microtubules ranges from r_p to r_c, tau diffuses on the surface of the two approaching microtubules and has the possibility to form a cross-bridge. The average time, T_C, during which microtubules crosses the distance r_c–r_p, is:

$$T_C = \frac{\left(r_p - r_c\right)^2}{D_{MT}} = \frac{4\pi\eta\left(r_p - r_c\right)^2 L}{K_B T ln\left(\frac{L}{2R}\right)} \quad (2)$$

We thus need to consider the average time, T_{dimer}, which is required for the formation of a tau cross-bridge when two microtubules are sufficiently close to each other. T_{dimer} depends on the diffusion constant of tau on microtubules, D_{tau}; the microtubule length and radius, $L$and R; the number of tau molecules per microtubule, N_{tau} and the size of the interacting domain of tau (r~r_c). We then obtain after some algebra:

$$T_{dimer} \sim \left(\frac{L}{N_{tau} r_c}\right)\left(\frac{2\pi R}{r_c}\right)^2\left(\frac{{r_c}^2}{D_{tau}}\right) \quad (3)$$

To efficiently prevent microtubule bundling, the time required to form tau cross-bridges in between approaching microtubules, T_{dimer}, has to be shorter the that time required for microtubules to cross the distance r_p–r_c, T_c.

Thus, at the limit, $T_{dimer} < T_C$:

$$\frac{L}{N_{tau}} < \frac{D_{tau}}{\left(\frac{(2\pi R)^2}{r_c}\right)} \frac{4\pi\eta\left(r_p - r_c\right)^2}{K_B T \ln\left(\frac{L}{2R}\right)} \qquad (4)$$

The number of tau proteins requires to prevent microtubule bundling is then defined by the following equation:

$$\frac{N_{tau}}{L} > \frac{D_{MT}\left(\frac{(2\pi R)^2}{r_c}\right)}{D_{tau}\left(r_p - r_c\right)^2}, \qquad (5)$$

Many experimental values are lacking to obtain a quantitative estimation of the critical tau:tubulin molar ratio required to prevent bundling. For example, the transverse diffusion constant of parallel microtubules in axons is unknown. In addition, the number of tau cross-bridges necessary for blocking microtubule diffusion remains also to be estimated. Due to these limitations, we only used scaling laws for the analytical approach and normalized values of the diffusion coefficient in the numerical approaches.

Critical tau:tubulin ratio in the polymer brush model:

In the polymer brush model (Brittain and Minko, 2007) , for a polymer of radius r_g, here that of tau, the minimal number of polymer molecules which allows an effective steric hindrance scales like:

$$\frac{2\Pi(R+r_G)}{2r_G} \quad (6)$$

for a microtubule cylinder of radius R. As there are 1650 tubulin dimers per µm of microtubules and by using r_g = 12.5 nm and R = 12.5 nm. The result leads to about 1 tau molecule for 6 tubulins.

Description of the numerical simulation:

The numerical simulation used in this study is an adaptation of a previous model that we developed to describe the diffusion of mRNAs on microtubules (Chernov et al., 2009). N microtubules were allowed to diffuse in a square area as indicated in figure 5. At the beginning of the simulations, microtubules were homogeneously distributed in a defined square area. Simulations were then performed using at least 90 000 iterations. For each iteration, the microtubules move in random direction by 0.3 nm step, which corresponds to an explored area of 27 000 nm^2 at the end of the simulation. In a similar way, tau diffuses in 2D on the surface of cylindrical microtubule with a relative diffusion coefficient, D_{tau}/D_{MT},. Using equation (1) with L = 500 nm, R = 12.5 nm, T = 37°C and η = 0.044 Pa.s for molecules of few hundreds of nanometers in the cytoplasm of HeLa cells (Kalwarczyk et al., 2011), D_{MT} = 4.6 10^{-2} µm²/s. As D_{Tau}, the measured diffusion constant of tau along microtubules, is about 0.15 µm²/s, D_{tau}/D_{MT} = 3.2. However, the viscosity of axoplasm is not known and axonal microtubules can be longer than 500 nm (D_{tau}/D_{MT} = 15 for L = 4 µm). D_{tau}/D_{MT} ratios ranging from 2 to 20 were then used for the numerical simulations (see figure 5).

When the distance separating two tau molecules at the surface of two different microtubules is less than r_c (15 nm unless stated otherwise), cross-bridges are formed and prevent microtubules from further approaching to each other. However, in the absence of tau-cross-bridges, when the separation distance between microtubules is shorter r_p (5 nm), interacting microtubules form irreversibly bundles due to short-ranged excluded volume interactions. The number of tau molecules, N, required to prevent microtubule bundling should be considered as arbitrary because many experimental parameters are lacking like the diffusion constant of microtubules. The point of such analysis is rather to understand the mechanism of microtubule separation and the impact of tau diffusion and the length of the tau cross-bridges on the efficiency of this mechanism.

References:

Brittain, W.J., and Minko, S. (2007). A structural definition of polymer brushes. Journal of Polymer Science Part A: Polymer Chemistry *45*, 3505-3512.

Chernov, K.G., Barbet, A., Hamon, L., Ovchinnikov, L.P., Curmi, P.A., and Pastre, D. (2009). Role of microtubules in stress granule assembly: microtubule dynamical instability favors the formation of micrometric stress granules in cells. The Journal of biological chemistry *284*, 36569-36580.

Hamon, L., Savarin, P., Curmi, P.A., and Pastre, D. (2011). Rapid assembly and collective behavior of microtubule bundles in the presence of polyamines. Biophysical journal *101*, 205-216.

Kalwarczyk, T., Ziebacz, N., Bielejewska, A., Zaboklicka, E., Koynov, K., Szymanski, J., Wilk, A., Patkowski, A., Gapinski, J., Butt, H.J., *et al.* (2011). Comparative analysis of viscosity of complex liquids and cytoplasm of mammalian cells at the nanoscale. Nano letters *11*, 2157-2163.

Yu, X., and Carlsson, A.E. (2004). Kinetics of filament bundling with attractive interactions. Biophysical journal *87*, 3679-3689.
